# Disseminated nontuberculous mycobacterial infection with cryptic immunodeficiency mimicking malignancy: a case report

**DOI:** 10.1186/s12890-022-02227-0

**Published:** 2022-11-29

**Authors:** Xiaochuan Sun, Ting Zhang, Hongli Sun, Xuefeng Sun

**Affiliations:** 1 Department of Pulmonary and Critical Care Medicine, Peking Union Medical College, Peking Union Medical College Hospital, Chinese Academy of Medical Science, No. 1 Shuaifuyuan, 100730 Beijing, People’s Republic of China; 2 Department of Laboratory Medicine, Peking Union Medical College, Peking Union Medical College Hospital, Chinese Academy of Medical Science, Beijing, People’s Republic of China

**Keywords:** Nontuberculous mycobacteria, *Mycobacterium Colombiense*, Anti-IFN-γ autoantibodies, Adult-onset immunodeficiency, Rituximab

## Abstract

**Background:**

Nontuberculous mycobacteria (NTM) usually invades vulnerable hosts. Disseminated NTM (dNTM) infection can affect nearly all organs and be easily misdiagnosed as metastatic carcinoma or other systemic diseases, especially in seemingly immunocompetent hosts. Identification of underlying immunodeficiency is critical for the diagnosis and treatment of dNTM. Adult-onset immunodeficiency (AOID) with anti-IFN-γ autoantibodies has recently been recognized as a crucial but frequently neglected risk factor for dNTM infection. Frequent relapses of infection are common in AOID patients despite appropriate anti-infective treatment and B-cell-depleting therapy has shown some promising results. Herein, we report a case of dNTM infection mimicking malignancy in an AOID patient who was successfully treated with rituximab.

**Case presentation:**

A middle-aged male presented with fever, productive cough, multifocal skin abscesses and multiple osteolytic lesions with pathological fractures. Chest CT revealed consolidation of the lingula while bronchoscopy showed a mass completely blocking the airway opening of the inferior lingual segment. Metagenomic next-generation sequencing and mycobacterial culture of skin pus and bronchoalveolar lavage fluid reported *Mycobacterium Colombiense*, confirming the diagnosis of dNTM infection. However, anti-NTM antibiotics alone failed to prevent disease relapse and progression. Further evaluation indicated undetectable serum IFN-γ concentration and high-titer autoantibodies against IFN-γ, suggesting that AOID was the underlying reason for dNTM. Rituximab was added to treatment and successfully controlled the infection without relapse at one-year follow-up.

**Conclusion:**

We reported a rare case of disseminated *Mycobacterium Colombiense* infection manifested with pulmonary mass, pathological fracture and dermapostasis in a host with AOID. Our case demonstrated that AOID should be screened when patients get the episode of disseminated NTM infection particularly when other risk factors are excluded. Besides prolonged anti-NTM therapy, AOID-associated NTM infection should be treated with B-cell-depleting therapy to prevent recurrence.

## Background

Nontuberculous mycobacteria (NTM) are a group of weakly virulent microorganisms ubiquitous in the environment [1]. In recent years, infections caused by NTM have been increasingly diagnosed worldwide and are notoriously challenging to treat due to resistance to many common antibiotics and incurable immunocompromised status in hosts [2, 3]. Disseminated NTM (dNTM) infection encompasses a broad spectrum of clinical manifestations which can affect nearly all organs and are thus frequently misdiagnosed as metastatic carcinoma, connective tissue diseases or lymphoma, especially in previously healthy individuals [4]. Although an increasing number of dNTM cases without apparent risk factors were reported, many of them actually have immunodeficiency which is not identified due to the limitation of available or routine tests for evaluation of immune status [5, 6].

Th1-reponses, characterized by IFN-γ secretion, play a pivotal role in the activation of monocytes to establish effective defenses to intracellular pathogens including NTM [7]. Current studies have suggested that formation of neutralizing anti-IFN-γ autoantibodies can cause adult-onset immunodeficiency (AOID) and increase susceptibility to a group of opportunistic pathogens, prominently in elderly patients of Southeast and East Asian origin [8, 9]. Early diagnosis of AOID can be considerably challenging as it involves specific testing not routinely available and its clinical manifestations are not distinctive [4]. Patients with AOID commonly suffer progressive and severe infections despite prolonged antimicrobial therapy, resulting in unfavorable outcomes. Treatment targeting the abnormal autoantibodies may be effective, highlighting the need for increased awareness of this syndrome among clinicians [10, 11]. Therefore, we present a case of an apparently healthy patient with disseminated and refractory NTM infections who tested positive for neutralizing antibodies against IFN-γ and finally received anti-CD20 treatment.

## Case presentation

**Fig. 1:**
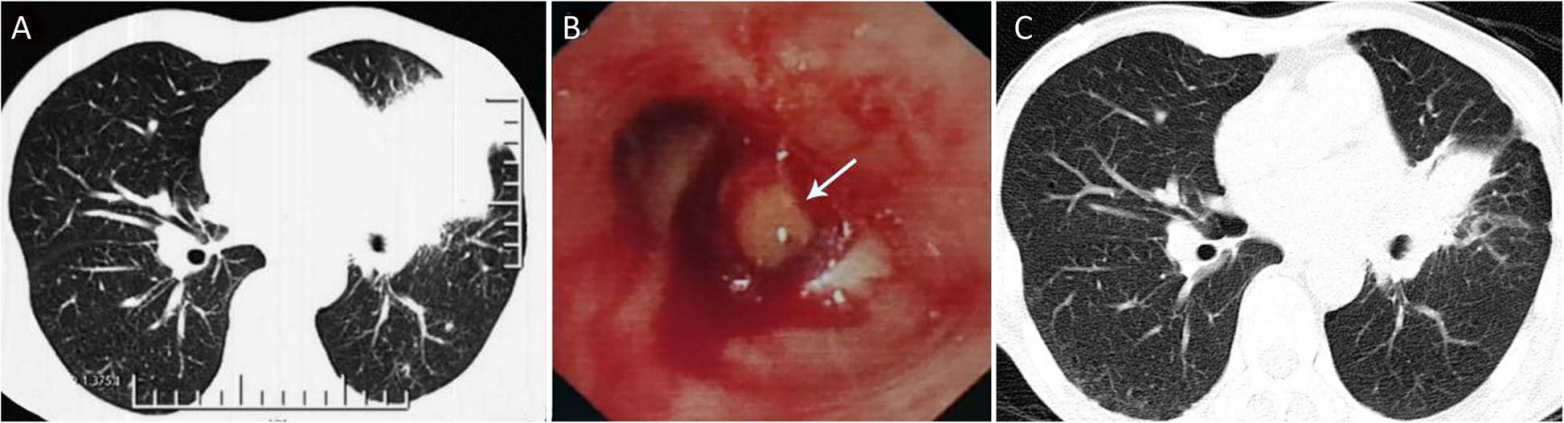
**A** Atelectasis in the lingula segment with obstructive pneumonia. **B** Endobronchial granulomatous mass obstructing the airway opening of the inferior lingual segment. **C** Partly absorbed pulmonary lesion in the lingula segment after treatment with anti-NTM antibiotics and rituximab

**Fig. 2:**
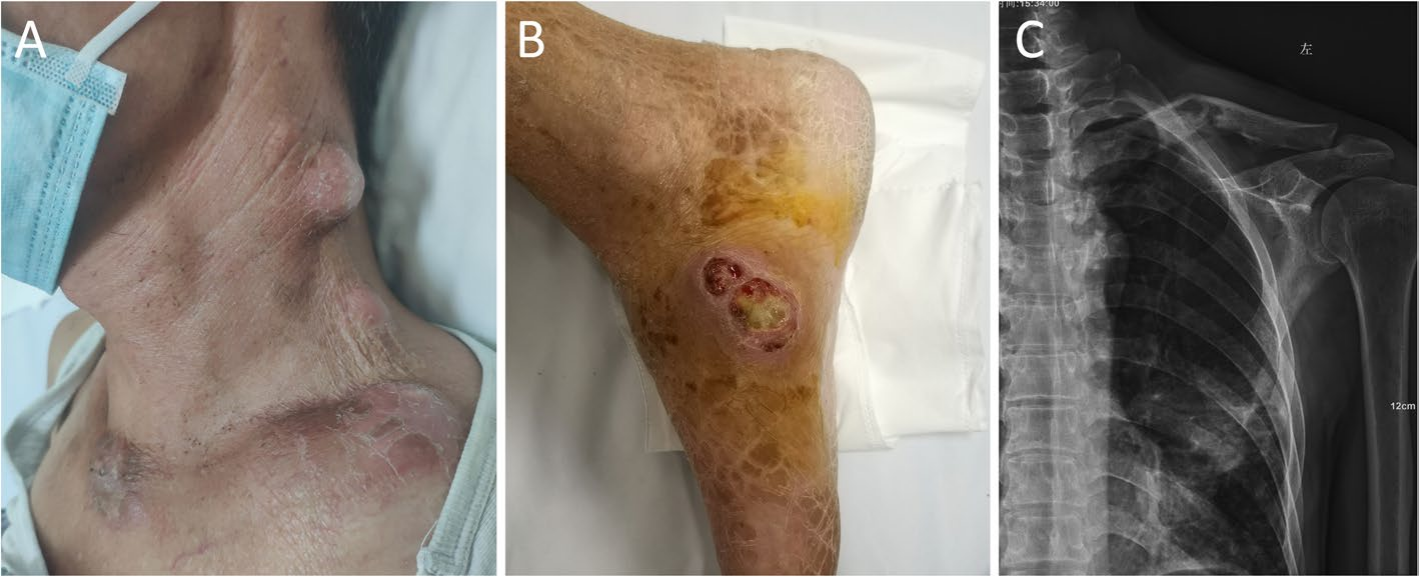
**A** Skin abscesses on the left neck. **B** Skin abscess on the right leg. **C** Osteolytic lesions with pathological fractures of the left clavicle

A 59-year-old male presented with 5 months of intermittent fever and productive cough. Laboratory findings showed significant elevation of white blood cell count (18.5 × 10^9^/L) and C-reactive protein (147 mg/L). Chest CT scan revealed consolidation of the lingula, mediastinal lymphadenopathy and left-sided pleural effusion (Fig. 1A). Bronchoscopy showed a mass completely blocking the airway opening of the inferior lingual segment (Fig. 1B). Transbronchial biopsy revealed pathological finding of granulomatous inflammation. Predominant neutrophils were observed in bronchoalveolar lavage fluid but all cultures were negative. The patient was treated with antibiotics including levofloxacin, ceftazidime, meropenem and vancomycin, but with no effect. Concurrently, the patient suffered an episode of herpes zoster infection. One month before admission, the patient developed multiple skin abscesses which spontaneously burst and drained white-yellow pus on the neck, back, elbows and legs (Fig. 2A, B). Meanwhile, he suffered from progressive pain and swelling of bilateral wrists, elbows, and shoulders. Radiological findings demonstrated multiple osteolytic lesions in scapulae, clavicles and T10 vertebrae, with pathological fracture of the left clavicle (Fig. 2C). The patient used to be a heavy smoker with a family history of gastrointestinal malignancy and reported no exposure to illicit drugs or immunosuppressants.

After admission, needle aspiration from skin abscesses and repeated bronchoalveolar lavage fluid were sent for microscopy and culture. Xpert MTB/RIF assay was negative, but acid-fast smears of both samples were positive. Metagenomic next-generation sequencing (mNGS) using whole genome shotgun approach of skin pus reported *Mycobacterium Colombiense (M. Colombiense*), which was also confirmed by mycobacterial culture for 623 h, establishing the diagnosis of dNTM infection. The patient was negative for HIV. A standard anti-NTM therapy with oral azithromycin, rifampicin, ethambutol and intravenous amikacin was firstly initiated for three months but could not successfully prevent the relapse and progression of NTM infection despite good patient compliance. As anti-mycobacterial alone was less effective, more diagnostics were conducted to investigate the underlying reason of dNTM infection. T cell subset analysis showed apparently decreased B cell count, but normal CD4^+^ and CD8^+^ T cell counts. Serum immunoglobulin levels were within the normal range. Further evaluation indicated undetectable serum IFN-γ concentration and high-titer autoantibodies against IFN-γ, suggesting the diagnosis of adult-onset immunodeficiency (AOID) with defects in IFN-γ signaling. Rituximab was then given intravenously as two 1 g infusions separated by two weeks and aforementioned anti-NTM antibiotics were continued. Thereafter, neck abscesses were almost completely absorbed and lung lesions significantly reduced in size three months later (Fig. 1 C). Follow-up reported no relapse of infection one year later.

## Discussion and conclusions

NTM represents over 190 species and subspecies, some of which are conditional pathogens. *M. Colombiense* is a novel recognized member of *Mycobacterium Avium Complex (MAC)*, the most common slow-growing NTM [1]. Infection with *M. colombiense* is very rare and has been only reported in a few cases. However, in view of the inability of prior molecular methods to discriminate the species diversity comprising *MAC*, the real prevalence of *M. Colombiense* may have been underestimated [12, 13]. According to prior studies, *M. colombiense* is prone to cause pulmonary disease and lymphadenopathy, rarely affecting skin and bone [12, 13]. To our knowledge, this is the first report of disseminated *M. colombiense* infection presented with pulmonary mass, pathological fracture and dermapostasis.

Pulmonary *MAC* infection is typically insidious, with chronic cough usually productive of purulent sputum and occasional hemoptysis. Besides typical upper-lobe fibrocavitary or nodular bronchiectatic presentation on pulmonary CT, mass or mass-like consolidation mimicking cancer, as illustrated in this case, can also be encountered. As previously reported, the incidence of NTM pulmonary disease mimicking malignancy is 3.6%, which represents a small but meaningful ratio because the consequences of misdiagnosis can be lethal [14]. Microbiological evidence is critical for definitive diagnosis of NTM infection. However, conventional acid-fast staining cannot distinguish NTM from *M. tuberculosis* while culture is time-consuming and sometimes shows false-negative results. Recently, mNGS has been increasingly applied in detecting microorganisms due to its advantages of accurate and rapid species-level pathogen identification [15]. It also provided important clue for considering NTM disease in our case, suggesting its potential value in NTM diagnosis.

Skin involvement is common in disseminated MAC infection. The presentation of cutaneous lesions may vary greatly from patient to patient, including panniculitis, papules, nodules, granulomas, pustules and ulcerations. Lesions in multiple stages of development may co-exist. Morphological features are usually non-specific and skin biopsy is the gold standard for diagnosis [16]. Data regarding cutaneous disease in *M. Colombiense* infection is scarce and only one case has been reported before. The patient presented with an impetiginous rash with hard exfoliation on his cranial-facial region, different from the morphology and distribution of cutaneous lesions in our patient [17]. Further investigation is needed to identify the pattern of dermopathy in patients with *M. Colombiense* infection.

NTM infection of bone leading to osteolytic lesions is a rare condition and can be easily misdiagnosed as *M. tuberculosis* infection. Different from *M. tuberculosis* which primarily affects load-bearing joints such as the thoracolumbar spine, hips, and knees, NTM can affect any bone of the body, often presenting as multiple bone involvement. The most frequently involved sites include the vertebrae, sternum, clavicle, and ribs, followed by the femur and ilium. Pathological fracture has been previously reported in several dNTM cases, mainly related to *M. abscessus* and *M. intracellulare*, which is also a member of *MAC* [18, 19]. Furthermore, IFN-γ is important in the maintenance of the balance between osteoclasts and osteoblasts [18]. Therefore, the defective IFN-γ signaling may also contribute to the pathogenesis of osteolytic lesions in our patient.

As a typical opportunistic pathogen, NTM usually invades immunocompromised hosts. Identifying these underlying reasons of NTM infections is always crucial as many of them are modifiable and neglecting them can lead to unfavorable outcomes [5]. AOID in patients with neutralizing anti-IFN-γ autoantibodies is an important risk factor of NTM infection. This syndrome appeared for the first time in 2004 and has become an emerging medical issue recently, particularly in Southeast Asia [8]. The exact etiology of AOID remains elusive. Nearly all the patients to date are of Asian descent, implicating the involvement of a common genetic factor [20]. According to a previous study, unexpectedly high frequencies of two HLA alleles, DRB1*16:02 and DQB1*05:02, were found in AOID patients, suggesting a potential association between HLA polymorphism and the development of anti-IFN-γ autoantibodies [20]. Patients with AOID are susceptible to severe and refractory infections caused by opportunistic pathogens, especially NTM [21]. As presented in this case, reactivation of varicella-zoster virus infection is also common in AOID hosts. Based on a prior report, 71% AOID patients suffered from herpes zoster [20]. The diagnosis of this syndrome can be established in patients with infections caused by unusual intracellular pathogens and positive for the anti-IFN-γ autoantibody. According to prior reports in Taiwan and Thailand, the prevalence of AOID can be extremely high in otherwise healthy patients with dNTM [21]. However, as a rare immunodeficiency disorder primarily affecting elderly patients with an insidious onset and indolent clinical course, AOID can be easily missed. Additionally, the lack of distinctive clinical manifestations and involvement of specific testing not routinely available make its early recognition even more challenging. An average diagnostic delay of 1.6 years has been reported recently [4]. AOID should thus be routinely screened when patients get the first episode of NTM infection, particularly when other risk factors are excluded [5, 20].

Frequent recurrences of infection are common in AOID patients despite prolonged anti-infective therapy and good patient compliance [21]. Therefore, treatment targeting the underlying condition is necessary to achieve long-term control of infections, but currently no standardized approach has been widely accepted. While immunosuppressive therapy seems counterintuitive in patients with disseminated infections, B-cell-depleting therapy with rituximab has shown promising results in several small studies [10, 21]. Consistent to these prior reports, a favorable response to rituximab was observed in our case. Future studies with a large sample size and longer follow-up period are warranted to further investigate the effectiveness and safety of rituximab in treatment of dNTM associated with AOID.

In conclusion, we present an intriguing case of dNTM infection manifested with pulmonary mass, pathological fracture and skin abscesses mimicking metastatic malignancy in a patient with AOID caused by abnormal formation of anti-IFN-γ autoantibodies. Considering the substantial challenge in early diagnosis of AOID, it is crucial to increase awareness of this syndrome among clinicians. Additional studies are warranted to provide a better understanding of the pathogenesis, clinical course and treatment strategies of this disease.

## Data Availability

The datasets used and/or analysed during the current study are available from the corresponding author on reasonable request. X. S. will make the data available to readers.

## References

[1] Falkinham JO (2013). Ecology of nontuberculous mycobacteria–where do human infections come from?. Semin Respir Crit Care Med.

[2] Donohue MJ, Wymer L (2016). Increasing Prevalence Rate of Nontuberculous Mycobacteria Infections in Five States, 2008–2013. Ann Am Thorac Soc.

[3] Wu J, Zhang Y, Li J (2014). Increase in nontuberculous mycobacteria isolated in Shanghai, China: results from a population-based study. PLoS One.

[4] Wu UI, Wang JT, Sheng WH (2020). Incorrect diagnoses in patients with neutralizing anti-interferon-gamma-autoantibodies. Clin Microbiol Infect.

[5] Lake MA, Ambrose LR, Lipman MC, Lowe DM (2016). ’"Why me, why now?“ Using clinical immunology and epidemiology to explain who gets nontuberculous mycobacterial infection. BMC Med.

[6] Albert-Vega C, Tawfik DM, Trouillet-Assant S (2018). Immune Functional Assays, From Custom to Standardized Tests for Precision Medicine. Front Immunol.

[7] Abe Y, Fukushima K, Hosono Y (2020). Host Immune Response and Novel Diagnostic Approach to NTM Infections. Int J Mol Sci.

[8] Browne SK, Burbelo PD, Chetchotisakd P (2012). Adult-onset immunodeficiency in Thailand and Taiwan. N Engl J Med.

[9] Aoki A, Sakagami T, Yoshizawa K (2018). Clinical Significance of Interferon-γ Neutralizing Autoantibodies Against Disseminated Nontuberculous Mycobacterial Disease. Clin Infect Dis.

[10] Browne SK, Zaman R, Sampaio EP (2012). Anti-CD20 (rituximab) therapy for anti-IFN-γ autoantibody-associated nontuberculous mycobacterial infection. Blood.

[11] Hong GH, Ortega-Villa AM, Hunsberger S (2020). Natural History and Evolution of Anti-Interferon-γ Autoantibody-Associated Immunodeficiency Syndrome in Thailand and the United States. Clin Infect Dis.

[12] Gosal J, Lee BC (2018). A case report of fatal disseminated Mycobacterium colombiense infection in a renal transplant recipient. Transpl Infect Dis.

[13] Poulin S, Corbeil C, Nguyen M (2013). Fatal Mycobacterium colombiense/cytomegalovirus coinfection associated with acquired immunodeficiency due to autoantibodies against interferon gamma: a case report. BMC Infect Dis.

[14] Hong SJ, Kim TJ, Lee JH, Park JS (2016). Nontuberculous mycobacterial pulmonary disease mimicking lung cancer: Clinicoradiologic features and diagnostic implications. Med (Baltim).

[15] Abe  Y, Fukushima K, Hosono Y (2020). Host Immune Response and Novel Diagnostic Approach to NTM Infections. Int J Mol Sci.

[16] Kollipara R, Richards K, Tschen J (2016). Disseminated Mycobacterium avium Complex With Cutaneous Lesions. J Cutan Med Surg.

[17] Inagaki Y, Ito T, Kato T (2018). Disseminated Cutaneous Infection of Mycobacterium colombiense in a Patient with Myelodysplastic Syndrome. Intern Med.

[18] Tang M, Huang J, Zeng W (2021). Retrospective Analysis of 10 Cases of Disseminated Nontuberculous Mycobacterial Disease with Osteolytic Lesions. Infect Drug Resist.

[19] Moral MZ, Desai K, Arain AR (2019). Mycobacterium abscessus-associated vertebral osteomyelitis in an immunocompetent patient: a rare case report and literature review. Spinal Cord Ser Cases.

[20] Chi CY, Chu CC, Liu JP (2013). Anti–IFN-γ autoantibodies in adults with disseminated nontuberculous mycobacterial infections are associated with HLA-DRB1*16:02 and HLA-DQB1*05:02 and the reactivation of latent varicella-zoster virus infection. Blood.

[21] Chi CY, Lin CH, Ho MW (2016). Clinical manifestations, course, and outcome of patients with neutralizing anti-interferon-γ autoantibodies and disseminated nontuberculous mycobacterial infections. Med (Baltim).

